# Effects of alternating current stimulation on the healthy and diseased brain

**DOI:** 10.3389/fnins.2015.00391

**Published:** 2015-10-27

**Authors:** Aini Ismafairus Abd Hamid, Carolin Gall, Oliver Speck, Andrea Antal, Bernhard A. Sabel

**Affiliations:** ^1^Department of Biomedical Magnetic Resonance, Institute for Experimental Physics, Otto-von-Guericke University MagdeburgMagdeburg, Germany; ^2^Department of Neurosciences, School of Medical Sciences, Universiti Sains MalaysiaKubang Kerian, Malaysia; ^3^Institute of Medical Psychology, Otto-von-Guericke University MagdeburgMagdeburg, Germany; ^4^Leibniz Institute for NeurobiologyMagdeburg, Germany; ^5^Center for Behavioral Brain SciencesMagdeburg, Germany; ^6^German Center for Neurodegenerative Disease (DZNE)Magdeburg, Germany; ^7^Department of Clinical Neurophysiology, University Medical Center, Georg-August UniversityGoettingen, Germany

**Keywords:** transcranial alternating current stimulation, transorbital alternating current stimulation, oscillation, EEG, synchronization

## Abstract

Cognitive and neurological dysfunctions can severely impact a patient's daily activities. In addition to medical treatment, non-invasive transcranial alternating current stimulation (tACS) has been proposed as a therapeutic technique to improve the functional state of the brain. Although during the last years tACS was applied in numerous studies to improve motor, somatosensory, visual and higher order cognitive functions, our knowledge is still limited regarding the mechanisms as to which type of ACS can affect cortical functions and altered neuronal oscillations seem to be the key mechanism. Because alternating current send pulses to the brain at predetermined frequencies, the online- and after-effects of ACS strongly depend on the stimulation parameters so that “optimal” ACS paradigms could be achieved. This is of interest not only for neuroscience research but also for clinical practice. In this study, we summarize recent findings on ACS-effects under both normal conditions and in brain diseases.

## Introduction

Patients may suffer from damage to the central nervous system because of a variety of diseases such as stroke or traumatic brain injury (TBI), neurodegeneration of the brain or the retina, or genetic predispositions. Depending on which areas of the brain are affected, this will lead to deficits in motor control, visual perception, language, attention, or memory deficits. These dysfunctions usually lead to substantial limitations in performing daily life activities, which may severely impact their quality of life (Dimyan and Cohen, 2011; Gall et al., 2011; Shah et al., 2013). There is strong evidence that functional recovery occurs due to effective and intensive therapy beyond the spontaneous, natural recovery (Dimyan and Cohen, 2011; Hamilton et al., 2011; Sabel et al., 2011b; Shah et al., 2013). In addition to more established methods of behavioral therapy, other options such as invasive and non-invasive brain stimulation methods have been proposed as promising possibilities among therapeutic options, with each technique offering its own advantages and disadvantages (Nizard et al., 2012; Nardone et al., 2014).

Behavioral therapy is believed to recover some of the functions mediated by surviving neurons or networks (Dobkin, 2005; Sabel et al., 2011b; Perrey, 2013). It involves training (rehabilitation) programs where a repetitive, behavioral tasks is carried out to repetitively stimulate brain areas and thus promote neuroplastic changes (Musso, 1999; Sabel and Kasten, 2000; Dobkin, 2005; Breier et al., 2009; Mang et al., 2013; Perrey, 2013). Cognitive training programs, such as motor skill and strength training (Jensen et al., 2005), vision restoration training (Kasten et al., 1998, 2006; Sabel and Gudlin, 2014), language comprehension training (Rogde et al., 2013), and neuropsychological training of activities of daily living (Ávila et al., 2004), are safe and effective rehabilitation methods which can induce functional recovery.

Invasive brain stimulation methods, such as deep brain stimulation, represent another type of treatment that involves surgical procedures (Vedam-Mai et al., 2014). Such invasive methods are considered primarily if other conventional therapeutic measures have been ineffective (Nizard et al., 2012). In contrast, non-invasive brain stimulation (NIBS) methods, recently developed alternatives do not involve surgery and include different methods such as transcranial magnetic stimulation (TMS), transcranial direct current stimulation (tDCS), transcranial, or transorbital alternating current stimulation (tACS), and transcranial random noise stimulation (tRNS). They are realatively inexpensive techniques requiring no surgery and have the potential to directly influence cortical activity and indirectly influence subcortical activity, to induce neuroplastic changes in the brain (Nardone et al., 2014). Interest has recently developed in the potential of NIBS, notably transcranial electrical stimulation (tES) (i.e., tDCS, tACS, and tRNS) to improve brain function or cognitive abilities in the healthy and diseased brain through distinct physiological mechanisms that vary according to the type of tES (Miniussi et al., 2013; Filmer et al., 2014; Santarnecchi et al., 2015). Essentially, tES methods are associated with the application of weak electrical currents that are delivered to the targeted brain area via electrodes placed to the scalp. The most frequently applied tES method is tDCS. The probable mechanism of tDCS is the modulation of neuronal membrane potentials which depend on the polarity of the stimulation, with anodal tDCS inducing membrane depolarization and cathodal tDCS inducing membrane hyperpolarization (Creutzfeldt et al., 1962; Bindman et al., 1964). More recently, tRNS and tACS have also been suggested as potential tES methods for modulating brain activity which involves the application of random electrical frequencies over the cortex (Terney et al., 2008; Miniussi et al., 2013; Santarnecchi et al., 2015). In contrast, tACS modulates ongoing neural oscillation at specific frequencies (Miniussi et al., 2013; Santarnecchi et al., 2015). In addition to tES methods, transorbital ACS also offers the potential to modulate ongoing neuronal activity at specific frequencies primarily in the visual system (Gall et al., 2010, 2011; Fedorov et al., 2011; Sabel et al., 2011a; Schmidt et al., 2013; Bola et al., 2014). Although, all NIBS methods have the potential to induce alterations in cortical plasticity, in this review, we would like to focus on the effects of transcranial and transorbital ACS in the brain. We chose this focus because the transcranial and transorbital ACS techniques offer the advantage of inducing direct cortical alterations in the intrinsic neural oscillation. Therefore, the aim of this review is to summarize recent knowledge related to the effects of ACS in the healthy and diseased brain.

## Basic principles of alternating current stimulation

**Figure 1:**
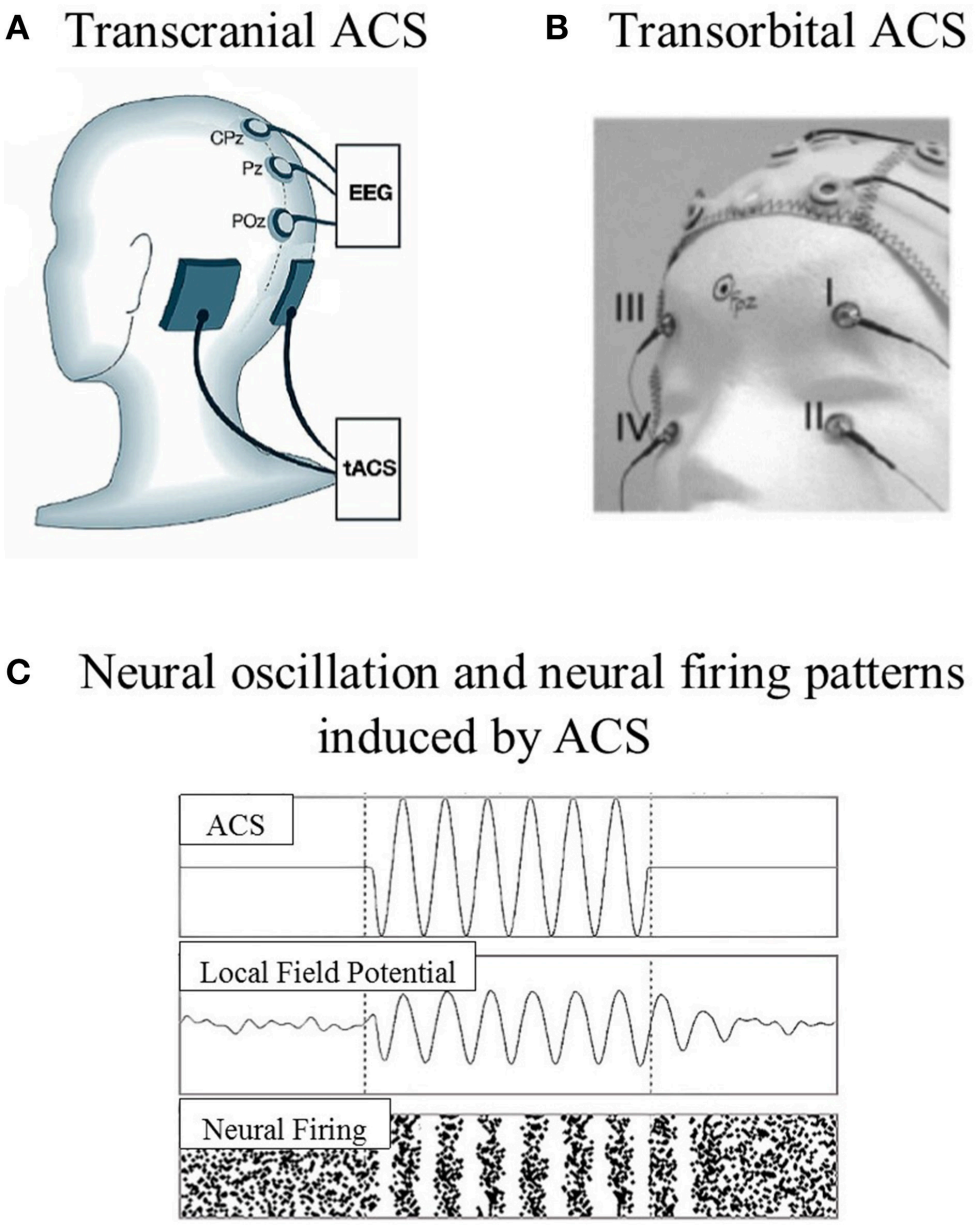
**Examples of stimulation electrode placements and neural oscillation. (A)** Transcranial ACS: electrodes were placed over the parieto-occipital region [P09 (target) and PO10 (reference), according to the 10–10 system]. **(B)** Transorbital ACS: four stimulation electrodes were positioned at or near the eyeballs (with eyes closed), and one electrode was positioned at the occipital pole as the reference electrode. **(C)** The model prediction of neural oscillation and neural firing patterns induced by ACS (adapted from Zaehle et al., 2010; Gall et al., 2011; Battleday et al., 2014).

ACS is a non-invasive stimulation technique that has been less intensively studied than tDCS and TMS approaches. ACS shares the same device and montage as tDCS, but uses different current wave forms (Zaghi et al., 2010). The waveform of the stimulation changes cyclically over time, with either sinus pulses or square pulses that penetrate the skull through the electrodes placed over the surface of the scalp or are transmitted through the eye and optic nerve to the brain (Zaghi et al., 2010; Gall et al., 2011). The stimulation electrodes can be placed according to the target location, which is known as transcranial placement (e.g., Zaehle et al., 2010), or near the eyeballs, which is known as transorbital placement (e.g., Gall et al., 2011; see Figure 1). The effects of ACS at the neuronal level highly depend on the parameters used, i.e., current density, frequency range, electrode size, and the location of the stimulation electrode.

## Mechanism of action

During ACS a specific frequency, or frequency band (e.g., tRNS) is applied. Most of the applied stimulation frequencies are within the human EEG frequency range (for a review see, Miniussi et al., 2012). However, more recently frequencies up to 5 kHz were also used (Chaieb et al., 2014).

Many studies suggest that ACS can entrain cortical oscillations. In 2008, Kanai et al. (2008) indicated that tACS entrains specific EEG frequency bands inducing local oscillatory activity in a stimulated brain area. Pogosyan et al. (2009) showed that tACS induced an increase in the EEG coherence at the beta frequency in the contralateral motor cortex and Zaehle et al. (2010) reported an increase in EEG spectral power after tACS involving the individual alpha frequency range in particular, as compared to the spectral power before ACS at baseline or during sham stimulation. This finding highlights the possible link between inherent frequency-specific oscillations and specific brain functions. Generally, speaking, the brain seems to mirror the frequencies induced from the outside by the stimulation, i.e., a 10 Hz stimulation will lead to a 10 Hz oscillatory response as measured by the EEG.

ACS-induced after-effects are assumed to arise from synaptic-level processes (Nardone et al., 2014). Synaptic plasticity refers to the ability of synapses to modify transmission efficacy or strength due to experience over time (Citri and Malenka, 2008). Synaptic plasticity not only plays an important role during early development but is also involved in brain functional recovery (Citri and Malenka, 2008). The two principle mechanisms that operate at the synaptic level are long-term potentiation (LTP) (i.e., synchronous signal transmission resulting in an increase in synaptic strength) and its opposite, long-term depression (LTD) (Citri and Malenka, 2008; Zaehle et al., 2010). In the healthy brain, the neuronal network ideally operates in a rather synchronous fashion. However, in patients with neurological disorders, alterations of the neuronal network and loss of brain cells and their connections may cause disturbances of synchronous firing (“desynchroniziation”); therefore, neuronal resynchronization is the goal to achieve functional improvement.

An ACS frequency that matches the endogenous frequency could entrain network oscillations (Zaehle et al., 2010; Herrmann et al., 2013; Reato et al., 2013; Battleday et al., 2014). For example, Zaehle et al. (2010) applied tACS to the simple spiking neuronal network at 10 Hz, which probably successfully increased the synapse strength. Recently, Vossen et al. (2015) reported increased EEG alpha power after tACS when the driven frequencies were at or above the individual alpha frequency (IAF) but not below IAF. Comparing EEG alpha power between phase continuity conditions (i.e., short, phase-continuous condition; long, phase-continuous condition; and long, phase-discontinuous condition) after tACS revealed no significant difference. Therefore, the after-effects of tACS were independent of phase continuity. Phase-locked alpha oscillation during tACS was not observed offline. From these findings, the authors proposed that tACS induced short term plasticity in the alpha activity rather than entrainment activity.

In addition, with regard to the physiological mechanism of tACS in humans reported by Zaehle et al. (2010) and Vossen et al. (2015), evidence was also provided by *in vivo* and *in vitro* animal studies performed by Fröhlich and McCormick (2010), Reato et al. (2010), Ozen et al. (2010), and Ali et al. (2013). Fröhlich and McCormick (2010) applied ACS intracranially in ferret cortical slices. Slow oscillations (physiological activity; i.e., up and down states) were induced spontaneously in *in vitro* slice preparations and remained preserved in *in vivo*-like artificial cerebrospinal fluid. The findings from recorded multi-unit activity revealed that AC fields were able to modulate the neuronal membrane voltage (i.e., up and down states). The AC fields applied at various frequencies revealed potential entraining slow oscillations, a stimulation frequency close to the endogenous frequency induced slow oscillations that were more periodic. However, Fröhlich and McCormick (2010) demonstrated only the effect of weak ACS when the stimulation electrode was placed intracranially. The question is whether the effect on neural activity remains the same when weak ACS penetrates the skull. To answer this question, Ozen et al. (2010) delivered ACS at a different frequency range (0.8–1.7 Hz) on the surface of the skull and simultaneously recorded intracranial neural activity in anesthetized rats. The authors demonstrated that ACS entrained 20 and 16% units in the cortical and hippocampal areas, respectively. The percentage of phase-locked neurons induced by ACS was intensity-dependent. The intensities that effectively phase-locked the spikes induced intracellular polarization values of 2–3 mV. An intracranial electrical field as low as 1 V/m was enough to synchronize neuronal spiking to an applied sinusoidal current. Apart from that, it was suggested that the value of stimulation intensity affected the number of spiking neurons recruited. They reported increased spiking activity with increased stimulation intensity. Reato et al. (2010) stimulated the CA3 region of rat hippocampal slices using ACS. The authors used 20 μM carbachol to induce neural oscillations in the gamma frequency band (25–35 Hz) in the CA3 region of the hippocampus. The recorded local field potentials showed that ACS modulated the oscillatory activity in a frequency-dependent manner. The application of ACS at a low frequency (7 Hz, 1 V/m) significantly modulated gamma power. This was induced by low frequency ACS, suggesting that it reflected changes in firing rate within the stimulation cycles. However, a net zero change in the firing rate of the gamma cycles (i.e., symmetric power modulation) indicated a rate-limiting property of the excitatory-inhibitory loop.

Additionally, the application of electrical stimulation at the endogenous frequency and at low amplitude (0.2 V/m) also revealed increased coherence of the spiking time. Based on these results, the authors suggested that ACS is able to modulate the firing rate, spiking time and excitation-inhibition balance in neurons. Ali et al. (2013) later demonstrated how ACS modulates both time and space dynamics in large-scale network dynamics of spiking neurons. Multi-unit activity was recorded in anesthetized ferrets to verify the findings obtained from the computational model. The results showed that tACS effectively entrained neuronal oscillation when the stimulation frequency was close to the intrinsic frequency of the networks (3 Hz). Moreover, the entrainment time was dependent on the onset phase of the oscillatory stimulation relative to the intrinsic network oscillation; the minimum entrainment time was observed at an onset phase of π. In line with previous results, the authors demonstrated that tACS modulates the neuronal networks in a phase- and frequency-dependent manner.

The above mentioned results demonstrate the possible physiological mechanisms that underlie tACS. However, the physiological mechanisms that underlie transorbital ACS still remain unclear. Recently, Foik et al. (2015) investigated the physiological mechanisms of transorbital ACS in rats. They found that neural activity originated from the retina and subsequently flowed through the visual pathway. They suggested that the increased synchronization of neural activity induced by transorbital ACS is likely a result of increased efficiency in the transfer of information and accelerated processing (Lesica et al., 2007; Wang et al., 2010) from the retina flowing through the lateral geniculate nucleus and subsequently, toward the cortical networks. By placing the stimulation electrodes around the eyeball, the authors implied that the plasticity induced by ACS is more effective when it starts in the early stages of visual processing, which is consistent with the results by Sergeeva et al. (2012). However, further experiments are still needed to address some issues that may induce the modulation of neural oscillation by transorbital ACS [for example, direct effects on the ongoing neural oscillation from other structures and the configurations of the stimulation electrodes (i.e., eye-eye and eye-neck) (Foik et al., 2015)].

In summary, the above mentioned physiological mechanisms suggest that transcranial ACS likely modulates neural oscillation focally in several brain areas, while transorbital ACS is likely effective in modulating the neural oscillation of visual networks. Indeed, both locations of the stimulation electrode (i.e., either transcranial placement or transorbital placement) have potential for modulating brain activity. However, future research is crucial to fully understand the mechanisms involved in both types of stimulation electrode placement, as no research comparing the physiological mechanisms of transcranial and transorbital ACS has been published to date.

Here, we highlighted evidence demonstrating that ACS can entrain neuronal network activity at different stimulation frequencies (Fröhlich and McCormick, 2010; Reato et al., 2010; Zaehle et al., 2010; Vossen et al., 2015) and that synchronized neural oscillation was effectively obtained when the stimulating frequency matched the intrinsic frequency of cortical networks (Fröhlich and McCormick, 2010; Reato et al., 2010; Zaehle et al., 2010). To summarize, ACS is thought to entrain brain oscillatory activity by increasing the number of neurons firing synchronously with the stimulation frequency, potentially inducing restoration after brain damage.

## Neuromodulation by ACS and effects on behavior

Because tACS tends to modulate ongoing oscillatory activity in the brain in a frequency-specific manner (Antal and Paulus, 2013; Herrmann et al., 2013) the question arises which stimulation parameters are critical for cortical modulation. This includes stimulation parameters such as stimulation duration, intensity, and electrode size. Examples of ACS studies in the healthy and diseased brain are listed in Table 1. Below, we summarize the previous findings of various experimental paradigms (e.g., motor, visual, somatosensory, and higher cognitive function).

**Table 1:** **Examples of ACS studies in the healthy and diseased brain**.

		**Article**	**Stimulation frequency**	**Stimulation intensity**	**Stimulation duration**	**Location of stimulation electrode**	**Location of reference electrode**	**Experiment**
Healthy Brain	Motor	Antal et al., 2008	1, 10, 15, 30, and 45 Hz	400 μA	5–10 min	Left motor cortex (4 × 4 cm)	Contralateral orbit (5 × 10 cm)	Motor learning
		Moliadze et al., 2010	140 and 250 Hz	1000 μA	10 min	Right first dorsal interosseous (FDI) (4 × 4 cm)	Forehead (14 × 6 cm)	Motor cortex excitability
		Feurra et al., 2011a	5, 10, 20, and 40 Hz	1000 μA	90 s	Left motor cortex (5 × 7 cm) and right parietal cortex (P4)	Pz according to 10–20 system (5 × 7 cm)	Motor cortex excitability
		Schutter and Hortensius, 2011	delta (1–3 Hz), theta (4–7 Hz), alpha (8–12 Hz) and beta (13–30 Hz)	1000 μA	10 min	Left motor cortex (C3) (5 × 7 cm)	Right motor cortex (C4) (5 × 7 cm)	Motor cortex excitability
		Chaieb et al., 2014	5 kHz	1000 μA	10 min	Left motor cortex (M1) (4 × 4 cm)	Contralateral orbit (7.5 × 6 cm)	EEG power spectra
	Voluntary movement	Pogosyan et al., 2009	5 and 20 Hz	580 ± 40 μA	10 periods of stimulation were randomly interspersed with 10 periods of no stimulation within 3 blocks for each session (total duration is not available)	Left motor cortex (4.5 × 3.2 cm)	Contralateral side of the neck (7.6 × 5.5 cm)	Motor performance
		Joundi et al., 2012	20 and 70 Hz	50 μA	Stimulation was randomly applied in 50% of trials (total duration is not available)	Left motor cortex (5 × 7 cm)	Ipsilateral shoulder (5 × 10 cm)	Motor performance
		Wach et al., 2013	10 and 20 Hz	1000 μA	10 min	Left motor cortex (M1) (5 × 7 cm)	Contralateral orbit (5 × 7 cm)	Motor performance
	Visual	Kanai et al., 2008	4, 8, 10, 12, 14, 16, 18, 20, 22, 24, 30, and 40 Hz	125, 250, 500, 750, and 1000 μA	10 s for each frequency/condition	Visual cortex- 4 cm above inion (4 × 4 cm)	Vertex (9 × 6 cm)	Phosephene perception
		Kanai et al., 2010	5, 10, 20, and 40 Hz	750 μA	5–8 min	Oz (7 × 5 cm)	vertex (7 × 5 cm)	Visual cortex excitibility
		Schutter and Hortensius, 2010	2, 10, and 20 Hz	1000 μA	10 s	Oz, Pz (5 × 7 cm)	Vertex (Cz), right shoulder (5 × 7 cm)	Retinal contribution to cortex
		Zaehle et al., 2010	(8–12 Hz)	1120 ± 489 μA	10 min	P09, PO10 (7 × 5 cm)	Oz (7 × 5 cm)	EEG alpha power
		Neuling et al., 2013	Individual alpha frequency	1500 μA	20 min	Oz according to 10–20 system (7 × 5 cm)	Vertex (Cz) according to 10–20 system (7 × 5 cm)	EEG alpha power
		Helfrich et al., 2014	10 Hz	1000 μA	20 min	Cz, Oz, according to 10-20 system (5 × 7 cm)	NA	EEG alpha power, EEG coherence
		Vossen et al., 2015	Individual alpha frequency	Individual stimulation intensity	3 s, 8 s	PO8-PO10, PO7-PO9 according to 10-10 system (5 × 7 cm)	Oz according to 10–20 system(5 × 7 cm)	EEG alpha power
	Somatosensory	Feurra et al., 2011b	2–70 Hz	1500 μA	5 s per trial	Right somatosensory cortex (3 × 4 cm)	Left posterior parietal cortex (5 × 7 cm)	Tactile sensation
	Higher cognitive functions	Polanía et al., 2012	6 Hz	1000 μA	14 ± 1.5 min	F3, P3 according to 10–20 system (5 × 5 cm)	Vertex (Cz) according to 10–20 system (5 × 5 cm)	Cognitive performance
		Sela et al., 2012	6.5 Hz	1000 μA	15 min	Left DLPFC (F3), left temporal (CP5) (5 × 5 cm)	left temporal (CP5), right temporal (CP6) (5 × 5 cm)	Cognitive performance
		Santarnecchi et al., 2013	5, 10, 20, and 40 Hz	750 μA	Duration of the task	Left middle frontal gyrus (5 × 7 cm)	Vertex (Cz) (5 × 7 cm)	Cognitive performance
		Lustenberger et al., 2015	10 and 40 Hz	2000 μA	30 min	F3, F4 according to 10–20 system	Vertex (Cz) (5 × 7 cm)	Cognitive performance
Diseased brain	Visual impairment	Fedorov et al., 2011	5–20 Hz	417 ± 156 μA	25–40 min of each session for 2 weeks (10 sessions in total)	Upper eyelids (two electrodes for each eye)	Wrist on the right hand	EEG power spectra, Visual field improvement
		Gall et al., 2011	5–30 Hz	Individual stimulation intensity (< 500 μA)	10–20 min for each eye/session for 10 days (10 sessions in total)	At or near eyeballs (two electrodes for each eye)	Occipital pole	Vision-specific quality of life, visual field improvement
		Sabel et al., 2011a	Individual stimulation frequency	Individual stimulation intensity	15 min for each eye/session for 010 days (10 sessions in total)	Upper eyelids (two electrodes for each eye)	Wrist on the right hand	EEG power spectra, Visual field improvement
		Schmidt et al., 2013	9–37 Hz	Individual stimulation intensity (< 500 μA)	25–40 min of each session for 10 days (10 sessions in total)	Superior and inferior to the eye (two electrodes for each eye)	Occipital pole	EEG power, Visual field improvement
		Bola et al., 2014	Individual stimulation frequency	Individual stimulation intensity	40 min of each session for 10 days (10 sessions in total)	At or near eyeballs (two electrodes for each eye)	NA	EEG power spectra, functional connectivity, coherence
	Parkinson disease	Brittain et al., 2013	Individual tremor frequency	2000 μA	10 min	Contralateral motor region (5 × 7 cm)	Ipsilateral (to tremor) shoulder (5 × 11 cm)	Phase alignments
		Krause et al., 2013	10 and 20 Hz	1000 μA	15 min	M1	Contralateral orbit	Motor performance, Cortico-muscular coupling

With regard to the primary motor cortex (Antal et al., 2008; Moliadze et al., 2010; Feurra et al., 2011a; Chaieb et al., 2014) it has been demonstrated that tACS selectively modulates brain oscillations depending on the stimulation parameters. In a study by Antal et al. (2008), ACS duration of 5–10 min with various frequencies (1, 10, 15, 30, and 45 Hz) and sham-stimulation were delivered over the left motor cortex in healthy subjects. The results suggest that 10 Hz tACS improves implicit motor learning slightly but does not alter cortical excitability, measured by eliciting motor-evoked potentials with TMS. In this study, for safety reasons, each subject was required to complete a questionnaire. Although, a few of the subjects experienced minor side-effects, such as light burning sensations under the electrode and mild headaches, none of them reported transient side-effects in 1–2 weeks follow-up. Feurra et al. (2011a) applied shorter stimulation duration of 90 s at different ACS frequencies (5, 10, 20, and 40 Hz) and sham stimulation over the left motor cortex in healthy subjects. Additionally, 20 Hz ACS and sham stimulation were delivered over the right parietal cortex for control stimulation, while 5 and 20 Hz were delivered on the right forearm (ulnar nerve) for peripheral control. The findings indicated that only ACS delivered at 20 Hz over the left motor cortex induced increased excitability as compared to other experimental conditions. With regard to the peripheral stimulation, no differences in the average amplitude of the compound muscle action potential were reported.

Schutter and Hortensius (2011) combined different ACS frequencies, i.e., delta (1–3 Hz), theta (4–7 Hz), alpha (8–12 Hz), and beta (13–30 Hz), over the left and right motor cortex in healthy subjects, to examine the associations between brain rhythms and cortical excitability. A linear regression analysis showed that the MEP amplitude in the primary motor cortex were modeled by theta and beta frequency range; therefore, the authors extended the study with combined frequencies, including theta-beta (5 and 20 Hz; for 5 min per frequency) and alpha-alpha (10 Hz for 10 min) over the primary motor cortex to examine the effects of the different designs. Cortical excitability increased after theta-beta frequency stimulation but not for alpha-alpha frequency stimulation. Stimulation in the theta and beta frequency ranges appears to contribute to increased cortical excitability; however, the specific contribution of each applied frequency is unknown.

Interestingly, Moliadze et al. (2010) found that higher frequency ACS (140 and 250 Hz, for 10 min) delivered over the representation of the right first dorsal interosseous muscle (M1) in healthy subjects can also modulate cortical excitability. Stimulation at 140 Hz increased M1 excitability, and that this after-effect persisted for up to 1 h in MEP amplitudes. Stimulation at 250 Hz increased MEP amplitudes during stimulation, but the increase in M1 excitability was sustained for only a short period (>5 min) after stimulation. No effects were reported at 80 Hz stimulation.

Chaieb et al. (2014) took one step further by applying tACS at 5 kHz to healthy subjects, even if it is clearly beyond the range of EEG. After application of ACS at 5 kHz for 10 min over the primary motor cortex a small reduction of delta activity was observed (Chaieb et al., 2014). This delta band oscillation reduction was proposed to be associated with performance enhancement (Wirth et al., 2011; Chaieb et al., 2014).

Voluntary movement studies (Pogosyan et al., 2009; Joundi et al., 2012; Wach et al., 2013) have reported the existence of task-specificity in brain oscillation. Go/no-go tasks (Joundi et al., 2012) performed after 20 and 70 Hz ACS over the left motor cortex in healthy subject indicated that opposite effects exist at these frequencies. Seventy Hertz tACS has been reported to increase performance in Go trials and to be ineffective during errors following no-go cues. However, 20 Hz tACS showed the opposite results: the participant's movements were slower during tACS, which was consistently reported in at least two studies (e.g., Pogosyan et al., 2009; Wach et al., 2013).

Apart from motor studies, ACS techniques are also used to explore brain rhythms and cortical excitability in visual processing (Herrmann et al., 2013). For example, Kanai et al. (2008) applied tACS to demonstrate the visibility of sensations of light (phosphenes) and the manipulation of cortical oscillations over the visual cortex under two light conditions (light and dark). Here, participants were stimulated by tACS at different intensities (125, 250, 500, 750, and 1000 μA) with different frequencies (4, 8, 10, 12, 14, 16, 18, 20, 22, 24, 30, and 40 Hz) in a random order. Under the light condition, perception of phosphenes was most effective in the beta frequency range (14–20 Hz), whereas in the dark condition, phosphenes were most effectively induced by alpha frequency stimulation (10–12 Hz). However, it remains to be seen if the phosphenes were evoked by cortical or retinal stimulation (or both). Recently, several studies attempted to address the crucial concern raised by Paulus (2010) regarding the potential contribution of retinal stimulation in visual cortex excitability (Schutter and Hortensius, 2010). Schutter and Hortensius (2010) delivered tACS at various frequencies (2, 10, and 20 Hz; 10 s duration of tACS; dimly lit room) in healthy subjects to examine the possibility of retinal influences on phosphenes. They reported more intense phosphenes during the frontalis-vertex montage than the occiput-vertex montage. Based on this result, they hypothesized the existence of volume conduction effects of the scalp. To support this hypothesis, a second experiment was performed to examine the voltage-related potentials from three different electrode placements (chantus, supra-orbital, and sub-orbital regions of the right eye). They reported that higher voltage-related potentials were measured with electrodes that were placed closer to the eye, therefore having greater potential for subjects to see more intense phosphenes. This evidence of voltage-related potentials demonstrates retinal contributions. However, supporting the subthreshold cortical stimulation hypotheses, Kanai et al. (2010) extended their studies to assess the modulation of the visual cortical excitability during tACS at various frequencies (5, 10, 20, and 40 Hz) that had been previously measured with TMS (measuring phosphene thresholds). Although ACS at 20 Hz increased the excitability of the visual cortex, no effect was observed for the other frequencies (5, 10, and 40 Hz). Hence, a contribution of retinal stimulation on the activation of the visual cortex remains possible.

In 2010, Zaehle et al. (2010) reported the first direct electrophysiological evidence of ACS effects on neuronal oscillatory activity in the visual cortex. In healthy subjects, ACS stimulation in the alpha frequency range (8–12 Hz) resulted in an increased EEG alpha power when compared to sham stimulation. In addition, Neuling et al. (2013) revealed that EEG after-effects depended on individual endogenous alpha power. Thus, tACS was only effective in influencing alpha power under conditions of low endogenous individual alpha power (open eyes) when compared to high endogenous individual alpha power (closed eyes). Recently, Vossen et al. (2015) observed increased EEG alpha power after tACS, particularly with long intermittent tACS (8 s) in comparison to short intermittent tACS (3 s). The EEG alpha power after tACS did not differ based on phase-continuity (short phase-continuous, long phase-continuous, and long phase-discontinuous). However, the phase-locking results showed that the synchronization of neural oscillation was not long-lasting, indicating that tACS induced plasticity in neuronal networks. This finding may also have relevance for the application of transorbital ACS, which will be discussed later.

Non-invasive ACS approaches have now demonstrated their potential as a therapeutic application for visual system damage, yet, so far there are only few studies (Chibisova et al., 2001; Fedorov et al., 2005, 2011; Gall et al., 2011; Sabel et al., 2011a). In early studies of transorbital ACS, small electrical currents were non-invasively applied to the eyeball area to stimulate visual pathway structures and the striate cortex (Chibisova et al., 2001; Fedorov et al., 2005). A few years after these initial studies, a clinical observational study on repetitive transorbital ACS (rtACS) was conducted that summarized data of 446 patients with optic nerve lesions (Fedorov et al., 2011). Here, a 10-day course of rtACS improved visual acuity and increased the size of visual fields in both eyes. During rtACS, frequency ranges are applied between the low alpha and flicker fusion frequencies (i.e., frequencies that induce a constant perception of light instead of distinct flickering phosphenes). EEG power spectra analysis revealed increased alpha power at occipital sites (Sabel et al., 2011a; Schmidt et al., 2013) indicating increased synchronization in the neural network after rtACS.

In a controlled randomized trial contrasting rtACS and sham-stimulation, vision restoration was observed only in the ACS-group, as indicated by a significant improvement in the visual field detection deficit, reduced reaction times, and increased visual acuity (Sabel et al., 2011a). Visual improvements were seen primarily in areas of residual vision, which is consistent with the residual vision activation theory (Sabel et al., 2011b). Ten-day application of rtACS resulted in long-term changes to impaired visual fields, as indicated by stable visual improvements observed at a 2 month follow-up. Moreover, improved visual functioning and vision-related quality of life in optic neuropathy after rtACS were reported (Gall et al., 2011).

Concerning the mechanisms of function beyond power spectra changes, rtACS effects in functional networks are beneficial for further understanding the underlying mechanisms of ACS. Bola et al. (2014) examined the alpha band functional connectivity and studied rtACS effects on perceptual functioning. Subjects with visual impairments due to optic nerve lesions had lower power of oscillatory activity, decreased strength of short- and long-range functional connectivity, and less clustered patterns of functional connectivity networks in comparison with a healthy control group. In those patients where either rtACS or sham treatment improved vision they observed increased alpha band functional connectivity and a modified the network topology indicating that synchronization within non-affected networks might also be relevant for vision restoration (Bola et al., 2014).

Besides being used in the visual system, ACS techniques also have the potential as a therapeutic application in Parkinson disease (PD). PD is a progressive movement disorder, that occurs when neurons in the brain, particularly in the substantia nigra begin to lose their function (Beitz, 2014; Gröger et al., 2014). Neurons in the substantia nigra are responsible for producing the neurotransmitter dopamine, which plays an important role for planning and regulating body movement (Arias-Carrión et al., 2010) and which mediate reward (Ilango et al., 2014) The reduced activity or death of neurons in the substantia nigra cause a reduction of dopamine content in the brain that influences a person's ability to control movement normally (Kourtidou et al., 2015). Since motor activity is associated with changes in beta frequency oscillations (Davis et al., 2012), beta-band stimulation has been suggested to modulate oscillatory activity in PD (Schnitzler and Gross, 2005). In 2013, Brittain et al. (2013) applied tACS over the motor cortex in patients diagnosed with tremor-dominant PD. Interestingly, tACS was most effective at the individual tremor frequency for inducing cortical phase cancellation (i.e., to suppress the resting tremor amplitude). Krause et al. (2013) studied the effects of 10 and 20 Hz ACS and sham-ACS in PD patients and healthy controls. In PD patients, after-effects appeared after 20 Hz tACS but not in the other conditions: the application of 20 Hz tACS reduced the beta band cortico-muscular coherence amplitude during isometric contraction and amplitude variation during fast finger tapping in PD patients, but not in healthy control subjects. In healthy subjects, effects found by Krause et al. (2013) differed from those seen in previous studies; possibly due to the higher age of participants and smaller differences in the applied tACS methodology (e.g., stimulation duration, (Krause et al., 2013) 15 min, (Wach et al., 2013) 10 min). However, the results revealed that transcranial beta-band ACS could modulate motor-cortical oscillation in PD patients, and that this technique has potential in PD therapy.

Previous findings of ACS are not restricted to motor and visual effects, but were also reported in the somatosensory modality (Feurra et al., 2011b). Feurra et al. (2011b) delivered tACS at 35 different frequencies ranging from 2 to 70 Hz in randomized order. Subjects were asked to rate the strength of tactile sensation. The findings indicated that transcranial ACS induced stronger tactile sensation at an alpha frequency range (10–14 Hz) and high gamma range (52–70 Hz) when compared to other stimulation frequencies.

Furthermore, tACS was observed to modify higher cognitive function (Polanía et al., 2012; Sela et al., 2012). Polanía et al. (2012) delivered 6 Hz tACS over the left prefrontal and parietal cortices (F3 and P3, respectively) with a relative 0° (“synchronized” condition) or 180° (“desynchronized” condition) phase difference compared to sham stimulation to investigate reaction times during a memory task in healthy subjects. In this study, tACS was administrated during a delayed letter discrimination task. Reaction times were longer in the desynchronized condition than in the sham condition. Sela et al. (2012) applied theta band transcranial ACS while investigating risk-taking behavior using a balloon analog risk task (BART). The BART is a computerized task designed to stimulate real-world risk behavior, such as gambling, that confers greater risk to wins and greater risk to losses. In the task, the participants are presented with balloons and given the opportunity to win a prize by obtaining a large number of points by pumping the balloon. The accumulated points depend on the decision made by participant to either keep pumping and risk explosion or stop pumping after each pump. In this study, tACS was applied at 6.5 Hz over the left or right dorsolateral prefrontal cortex (DLPFC). Healthy subjects were randomized into three different stimulation groups with different electrode positions: left DLPFC, right DLPFC, or sham stimulation (with left DLPFC or right DLPFC montage). No transcranial ACS effects were reported for the right DLPFC and sham conditions; however, sequential losses of the chance to win a prize increased during the tACS stimulation over the left DLPFC. The authors suggested that the subjects experienced a reduced ability to process and adjust their actions based on errors or negative feedback in this condition. The authors proposed that theta band ACS could modulate decision-making. Santarnecchi et al. (2013) applied transcranial ACS while performing Raven's Advances Progressive Matrices (RAPM) in healthy subjects. RAPM is a nonverbal intelligence test to determine the individual fluid intelligence, which is the ability to understand and solve problems. In the intelligence test, the stimulus features, approximately 3000 matrices with a mixture of different shapes, colors and orientations, were presented in the form of a 3 × 3 × 3 matrix. The subjects were given a maximum of 60 s to recognize the missing component that completed a pattern. The correct response could be deduced based on the type and number of analogical operations (matrices with one, two, or three relations and logic trials). During the intelligence test, the participants were stimulated by ACS at different frequencies (5, 10, 20, and 40 Hz) and sham stimulation in a random order. The stimulation electrodes were placed over the left middle frontal gyrus. Stimulation at 40 Hz was associated with improved performance (i.e., decreased response time), especially for more complex trials (logic trial), in comparison to sham and other stimulation frequencies. The authors proposed that gamma band stimulation could enhance fluid intelligence abilities in cognitive tasks. Lustenberger et al. (2015) applied ACS at 10 Hz and sham stimulation over the bilateral frontal cortex while performing the Torrance Test of Creative Thinking (TTCT) to determine the effect of 10 Hz on creative thinking in healthy subjects. The findings showed that 10 Hz transcranial ACS increased creativity index scores in comparison to sham stimulation. Improvements in individual creativity subscales (i.e., fluency, originality, elaboration, abstractness of titles and resistance to premature closures) were also observed for 10 Hz transcranial ACS relative to sham stimulation. Subsequently, to rule out possible effects of frequency-specific modulation of creativity, Lustenberger et al. (2015) extended their study in a second experiment. Here, the authors applied transcranial ACS at 40 Hz delivered over the bilateral frontal cortex while performing the TTCT. No improvements in the creativity index score were observed for 40 Hz transcranial ACS compared to sham stimulation. No significant differences in individual creativity subscales were observed for 40 Hz transcranial ACS compared to sham stimulation. Based on those two experiments, the authors proposed that the application of transcranial ACS at alpha band frequencies (10 Hz) could improve creative thinking.

As it was summarized above, evidence that ACS can modulate cortical oscillation has been shown in a number of studies. However, most of the studies focused on and reported electrophysiological findings based on the after-effects of the stimulation. Helfrich et al. (2014) successfully demonstrated the online electrophysiological effects of tACS using a visual task. In their study 20 min of 10 Hz tACS was delivered over the parietal-occipital cortex and concurrent EEG was recorded. tACS induced an increase of parieto-occipital alpha activity and synchronized cortical oscillation between intrinsic frequencies and entrainment frequencies. Interestingly, alpha power during stimulation was significantly correlated with the relative increase in alpha power after stimulation. This was interpreted as power enhancement induced by tACS, which outlasted the stimulation. Additionally, they showed that tACS modulates targeted detection performance in a phase dependent fashion of exogenous alpha oscillation.

## Safety issues

Previous studies demonstrated that non-invasive brain stimulation, including ACS, is safe with a fairly low risk of side-effects if used within the allowed safety limits (Nitsche et al., 2003, 2008; Poreisz et al., 2007; Bikson et al., 2009; Brunoni et al., 2011). To limit the probability of side effects and adverse implications, various safety limits have been defined, such as the highest current density, charge density, stimulation duration and stimulation frequency (Nitsche et al., 2003, 2008; Poreisz et al., 2007; Bikson et al., 2009). Only minor side effects have been reported by subjects, including a light itching sensation under the electrode or sponge during stimulation, tingling, a burning sensation, mild headache, nausea, and fatigue (Nitsche et al., 2003, 2008; Poreisz et al., 2007; Bikson et al., 2009; Brunoni et al., 2011). In the case of transorbital ACS, no serious side effects were observed during clinical trials (Gall et al., 2011; Sabel et al., 2011a). Although, the side effects of ACS are considered to be mild, the side effects of repetitive ACS application and even a single session of ACS have not been fully clarified. Therefore, future studies are needed to further investigate the safety of these methods, as few publications on this topic are currently available.

## General discussion

The biggest challenge for the progression of neuroprotection and neurorehabilitation is to maximize the benefits of the available treatment methods. A number of methods that offer the potential to enhance cognitive function, such as behavioral therapy (Ávila et al., 2004; Jensen et al., 2005; Kasten et al., 2006; Rogde et al., 2013; Sabel and Gudlin, 2014), deep brain stimulation (Nizard et al., 2012; Vedam-Mai et al., 2014), tDCS (Nitsche and Paulus, 2000; Antal et al., 2004; Liebetanz et al., 2009; Liu et al., 2014; Miller et al., 2015), tRNS (Terney et al., 2008; Chaieb et al., 2009; Fertonani et al., 2011; Romanska et al., 2015), tACS (Antal et al., 2008; Kanai et al., 2008; Pogosyan et al., 2009; Moliadze et al., 2010; Paulus, 2010; Schutter and Hortensius, 2010, 2011; Zaehle et al., 2010; Feurra et al., 2011a; Sela et al., 2012; Neuling et al., 2013; Wach et al., 2013; Chaieb et al., 2014) and transorbital ACS (Fedorov et al., 2005; Gall et al., 2011; Sabel et al., 2011a; Schmidt et al., 2013; Bola et al., 2014), are currently available. However, more recently, NIBS methods, especially tES (i.e., tDCS, tACS, and tRNS) (Miniussi et al., 2013; Filmer et al., 2014; Santarnecchi et al., 2015) and transorbital ACS (Fedorov et al., 2005; Gall et al., 2011; Sabel et al., 2011a; Schmidt et al., 2013; Bola et al., 2014) have offered clear benefits for neuroprotection and neurorehabilitation. Various studies have highlighted the potential of electrical brain stimulation to improve cognitive function through distinct physiological mechanisms. For example, tDCS induces changes in cortical excitability depending on the polarity of stimulation (Miniussi et al., 2013; Filmer et al., 2014; Santarnecchi et al., 2015), tRNS modifies cognitive performance depending on the applied frequency range (Miniussi et al., 2013; Santarnecchi et al., 2015) and transcranial and transorbital ACS modulate cortical excitability at specific frequencies (Antal et al., 2008; Antal and Paulus, 2013; Herrmann et al., 2013; Miniussi et al., 2013; Santarnecchi et al., 2015). Consequently, there is rapidly developing interest in implementing electrical brain stimulation techniques therapeutically to reduce cognitive impairment e.g., in patients with TBI.

In addition to NIBS, pharmacological approaches also have potential as treatment methods for cognitive deficits (Schmoll et al., 2003; Buga et al., 2012). For example, Buga et al. (2012) and Schmoll et al. (2003) applied pentylenetetrazole (PTZ) for a 25-day period in rats and demonstrated a potential benefit for inducing brain plasticity. A 25-day PTZ treatment was capable of generating new neurons (Schmoll et al., 2003; Buga et al., 2012). Buga et al. (2012) suggested that PTZ treatment can modulate changes in brain activity e.g., in epilepsy. However, the exact mechanisms of PTZ' action with regard to the neurogenesis are still unclear. Based on the potential benefits of repetitive PTZ application and NIBS, such as rtACS, in neuroprotection and neurorehabilitation, both methodologies have the potential to influence brain plasticity. However, NIBS, specifically transcranial and transorbital ACS are non-invasive applications, they can modify brain plasticity focally without involving any injections, and therefore probably with less side effects.

## Summary and future direction

The number of studies using NIBS techniques such as TMS and tDCS, have increased rapidly in the past 20 years. More recently, transcranial and transorbital ACS is a relatively new NIBS methods that can modulate brain rhythm activity has rapidly growing interest. The evidence presented indicates promising effects of ACS in various experiments, such as motor (Antal et al., 2008; Pogosyan et al., 2009; Moliadze et al., 2010; Feurra et al., 2011a; Schutter and Hortensius, 2011; Wach et al., 2013; Chaieb et al., 2014), visual (Fedorov et al., 2005; Kanai et al., 2008; Paulus, 2010; Schutter and Hortensius, 2010; Zaehle et al., 2010; Gall et al., 2011; Sabel et al., 2011a; Neuling et al., 2013; Schmidt et al., 2013; Bola et al., 2014), somatosensory (Feurra et al., 2011b) and higher cognitive functions (Polanía et al., 2012; Sela et al., 2012).

To date, only a limited number of studies have reported the beneficial effects of ACS in clinical populations, such as in patients with PD (Brittain et al., 2013; Krause et al., 2013) and with visual impairment (Fedorov et al., 2011; Gall et al., 2011; Sabel et al., 2011a; Bola et al., 2014). Despite the reported positive effects of ACS, less is known about the mechanisms and function reorganization. There may also be other mechanisms at work which are independent of neuronal oscillatory activity, such as blood flow coupling and brain metabolic activity changes. Therefore, more research is needed in order e.g., to discover the synaptic mechanisms involved and to find the optimal stimulation protocols and parameters not only in research but also in clinical practice. ACS has the potential to become one of the new options in the therapeutic field of brain stimulation with a fairly low risk of side-effects. For future studies, we suggest combining functional neuroimaging methods (such as functional magnetic resonance imaging) and ACS to investigate the immediate and after-effects. Until now only a limited number of combined experiments was reported using tDCS (Antal et al., 2011; Holland et al., 2011; Clark et al., 2012; Meinzer et al., 2014) and tRNS (Chaieb et al., 2009). This is a particularly interesting approach that can provide more information about the functional activation and interaction between brain regions and their modulation by ACS. Knowledge about the spatial (activation area), temporal and activation strength (signal intensity) effects in relevant brain regions would complement results from electrophysiological and behavioral measures.

## Author contributions

Substantial contributions to the conception or design of the work: AAH. Drafting the work or revising it critically for important intellectual content: AAH, CG, OS, AA. Final approval of the version to be published: CG, OS, AA; BS. Agreement to be accountable for all aspects of the work in ensuring that questions related to the accuracy or integrity of any part of the work are appropriately investigated and resolved: AAH, CG, AA.

### Conflict of interest statement

The Guest Associate Editor Dr. Henrich-Noack declares that, despite being affiliated to the same institution as authors Aini I. Abd Hamid; Carolin Gall; Oliver Speck; Bernhard A. Sabel, the review process was handled objectively. The authors declare that the research was conducted in the absence of any commercial or financial relationships that could be construed as a potential conflict of interest.

## References

[B1] Ali M. M. Sellers K. K. Fröhlich F. (2013). Transcranial alternating current stimulation modulates large-scale cortical network activity by network resonance. J. Neurosci. 33, 11262–11275. 10.1523/JNEUROSCI.5867-12.2013 23825429 PMC6618612

[B2] Antal A. Paulus W. (2013). Transcranial alternating current stimulation (tACS). Front. Hum. Neurosci. 7:317. 10.3389/fnhum.2013.00317 23825454 PMC3695369

[B3] Antal A. Boros K. Poreisz C. Chaieb L. Terney D. Paulus W. (2008). Comparatively weak after-effects of transcranial alternating current stimulation (tACS) on cortical excitability in humans. Brain Stimul. 1, 97–105. 10.1016/j.brs.2007.10.001 20633376

[B4] Antal A. Kincses T. Z. Nitsche M. A. Bartfai O. Paulus W. (2004). Excitability changes induced in the human primary visual cortex by transcranial direct current stimulation: direct electrophysiological evidence. Invest. Ophthalmol. Vis. Sci. 45, 702–707. 10.1167/iovs.03-0688 14744917

[B5] Antal A. Polania R. Schmidt-Samoa C. Dechent P. Paulus W. (2011). Transcranial direct current stimulation over the primary motor cortex during fMRI. Neuroimage 55, 590–596. 10.1016/j.neuroimage.2010.11.085 21211569

[B6] Arias-Carrión O. Stamelou M. Murillo-Rodríguez E. Menéndez-González M. Pöppel E. (2010). Dopaminergic reward system: a short integrative review. Int. Arch. Med. 3:24. 10.1186/1755-7682-3-24 20925949 PMC2958859

[B7] Ávila R. Bottino C. M. C. Carvalho I. A. M. Santos C. B. Seral C. Miotto E. C. (2004). Neuropsychological rehabilitation of memory deficits and activities of daily living in patients with Alzheimer's disease: a pilot study. Braz. J. Med. Biol. Res. 37, 1721–1729. 10.1590/S0100-879X2004001100018 15517089

[B8] Battleday R. M. Muller T. Clayton M. S. Cohen Kadosh R. (2014). Mapping the mechanisms of transcranial alternating current stimulation: a pathway from network effects to cognition. Front. psychiatry 5:162. 10.3389/fpsyt.2014.00162 25477826 PMC4237786

[B9] Beitz J. M. (2014). Parkinson's disease: a review. Front. Biosci. (Schol. Ed.) 6, 65–74. 10.2741/S415 24389262

[B10] Bikson M. Datta A. Elwassif M. (2009). Establishing safety limits for transcranial direct current stimulation. Clin. Neurophysiol. 120, 1033–1034. 10.1016/j.clinph.2009.03.018 19394269 PMC2754807

[B11] Bindman L. J. Lippold O. C. Redfearn J. W. (1964). The action of brief polarizing currents on the cerebral cortex of the rat (1) during current flow and (2) in the production of long-lasting after-effects. J. Physiol. 172, 369–382. 10.1113/jphysiol.1964.sp007425 14199369 PMC1368854

[B12] Bola M. Gall C. Moewes C. Fedorov A. Hinrichs H. Sabel B. A. (2014). Brain functional connectivity network breakdown and restoration in blindness. Neurology 83, 542–551. 10.1212/WNL.0000000000000672 24991030

[B13] Breier J. I. Juranek J. Maher L. M. Schmadeke S. Men D. Papanicolaou A. C. (2009). Behavioral and neurophysiologic response to therapy for chronic aphasia. Arch. Phys. Med. Rehabil. 90, 2026–2033. 10.1016/j.apmr.2009.08.144 19969164 PMC3068866

[B14] Brittain J.-S. Probert-Smith P. Aziz T. Z. Brown P. (2013). Tremor suppression by rhythmic transcranial current stimulation. Curr. Biol. 23, 436–440. 10.1016/j.cub.2013.01.068 23416101 PMC3629558

[B15] Brunoni A. R. Amadera J. Berbel B. Volz M. S. Rizzerio B. G. Fregni F. (2011). A systematic review on reporting and assessment of adverse effects associated with transcranial direct current stimulation. Int. J. Neuropsychopharmacol. 14, 1133–1145. 10.1017/S1461145710001690 21320389

[B16] Buga A.-M. Vintilescu R. Balseanu A. T. Pop O. T. Streba C. Toescu E.. (2012). Repeated PTZ treatment at 25-day intervals leads to a highly efficient accumulation of doublecortin in the dorsal hippocampus of rats. PLoS ONE 7:e39302. 10.1371/journal.pone.0039302 22768071 PMC3387140

[B17] Chaieb L. Antal A. Pisoni A. Saiote C. Opitz A. Ambrus G. G.. (2014). Safety of 5 kHz tACS. Brain Stimul. 7, 92–96. 10.1016/j.brs.2013.08.004 24064065

[B18] Chaieb L. Kovacs G. Cziraki C. Greenlee M. Paulus W. Antal A. (2009). Short-duration transcranial random noise stimulation induces blood oxygenation level dependent response attenuation in the human motor cortex. Exp. Brain Res. 198, 439–444. 10.1007/s00221-009-1938-7 19649624

[B19] Chibisova A. N. Fedorov A. B. Fedorov N. A. (2001). Neurophysiological characteristics of compensation-recovery processes in the brain during rehabilitation of the neurosensory impairment of the visual and acoustic systems. Fiziol. Cheloveka 27, 14–21. Available online at: http://pubget.com/paper/11548421 11548421

[B20] Citri A. Malenka R. C. (2008). Synaptic plasticity: multiple forms, functions, and mechanisms. Neuropsychopharmacology 33, 18–41. 10.1038/sj.npp.1301559 17728696

[B21] Clark V. P. Coffman B. A. Mayer A. R. Weisend M. P. Lane T. D. R. Calhoun V. D.. (2012). TDCS guided using fMRI significantly accelerates learning to identify concealed objects. Neuroimage 59, 117–128. 10.1016/j.neuroimage.2010.11.036 21094258 PMC3387543

[B22] Creutzfeldt O. D. Fromm G. H. Kapp H. (1962). Influence of transcortical d-c currents on cortical neuronal activity. Exp. Neurol. 5, 436–452. 10.1016/0014-4886(62)90056-0 13882165

[B23] Davis N. J. Tomlinson S. P. Morgan H. M. (2012). The role of β-frequency neural oscillations in motor control. J. Neurosci. 32, 403–404. 10.1523/JNEUROSCI.5106-11.2012 22238075 PMC6621073

[B24] Dimyan M. A. Cohen L. G. (2011). Neuroplasticity in the context of motor rehabilitation after stroke. Nat. Rev. Neurol. 7, 76–85. 10.1038/nrneurol.2010.200 21243015 PMC4886719

[B25] Dobkin B. H. (2005). Clinical practice. Rehabilitation after stroke. N. Engl. J. Med. 352, 1677–1684. 10.1056/NEJMcp043511 15843670 PMC4106469

[B26] Fedorov A. B. Chibisova A. N. Tchibissova J. M. (2005). Impulse modulating therapeutic electrical stimulation (IMTES) increases visual field size in patients with optic nerve lesions. Int. Congr. Ser. 1282, 525–529. 10.1016/j.ics.2005.05.007

[B27] Fedorov A. Jobke S. Bersnev V. Chibisova A. Chibisova Y. Gall C.. (2011). Restoration of vision after optic nerve lesions with noninvasive transorbital alternating current stimulation: a clinical observational study. Brain Stimul. 4, 189–201. 10.1016/j.brs.2011.07.007 21981854

[B28] Fertonani A. Pirulli C. Miniussi C. (2011). Random noise stimulation improves neuroplasticity in perceptual learning. J. Neurosci. 31, 15416–15423. 10.1523/JNEUROSCI.2002-11.2011 22031888 PMC6703532

[B29] Feurra M. Bianco G. Santarnecchi E. Del Testa M. Rossi A. Rossi S. (2011a). Frequency-dependent tuning of the human motor system induced by transcranial oscillatory potentials. J. Neurosci. 31, 12165–12170. 10.1523/JNEUROSCI.0978-11.2011 21865459 PMC6623220

[B30] Feurra M. Paulus W. Walsh V. Kanai R. (2011b). Frequency specific modulation of human somatosensory cortex. Front. Psychol. 2:13. 10.3389/fpsyg.2011.00013 21713181 PMC3111335

[B31] Filmer H. L. Dux P. E. Mattingley J. B. (2014). Applications of transcranial direct current stimulation for understanding brain function. Trends Neurosci. 37, 742–753. 10.1016/j.tins.2014.08.003 25189102

[B32] Foik A. T. Kublik E. Sergeeva E. G. Tatlisumak T. Rossini P. M. Sabel B. A.. (2015). Retinal origin of electrically evoked potentials in response to transcorneal alternating current stimulation in the rat. Invest. Ophthalmol. Vis. Sci. 56, 1711–1718. 10.1167/iovs.14-15617 25650414

[B33] Fröhlich F. McCormick D. A. (2010). Endogenous electric fields may guide neocortical network activity. Neuron 67, 129–143. 10.1016/j.neuron.2010.06.005 20624597 PMC3139922

[B34] Gall C. Fedorov A. B. Ernst L. Borrmann A. Sabel B. A. (2010). Repetitive transorbital alternating current stimulation in optic neuropathy. NeuroRehabilitation 27, 335–341. 10.3233/NRE-2010-0617 21160123

[B35] Gall C. Sgorzaly S. Schmidt S. Brandt S. Fedorov A. Sabel B. A. (2011). Noninvasive transorbital alternating current stimulation improves subjective visual functioning and vision-related quality of life in optic neuropathy. Brain Stimul. 4, 175–188. 10.1016/j.brs.2011.07.003 21981853

[B36] Gröger A. Kolb R. Schäfer R. Klose U. (2014). Dopamine reduction in the substantia nigra of Parkinson's disease patients confirmed by *in vivo* magnetic resonance spectroscopic imaging. PLoS ONE 9:e84081. 10.1371/journal.pone.0084081 24416192 PMC3885536

[B37] Hamilton R. H. Chrysikou E. G. Coslett B. (2011). Mechanisms of aphasia recovery after stroke and the role of noninvasive brain stimulation. Brain Lang. 118, 40–50. 10.1016/j.bandl.2011.02.005 21459427 PMC3109088

[B38] Helfrich R. F. Schneider T. R. Rach S. Trautmann-Lengsfeld S. A. Engel A. K. Herrmann C. S. (2014). Entrainment of brain oscillations by transcranial alternating current stimulation. Curr. Biol. 24, 333–339. 10.1016/j.cub.2013.12.041 24461998

[B39] Herrmann C. S. Rach S. Neuling T. Strüber D. (2013). Transcranial alternating current stimulation: a review of the underlying mechanisms and modulation of cognitive processes. Front. Hum. Neurosci. 7:279. 10.3389/fnhum.2013.00279 23785325 PMC3682121

[B40] Holland R. Leff A. P. Josephs O. Galea J. M. Desikan M. Price C. J.. (2011). Speech facilitation by left inferior frontal cortex stimulation. Curr. Biol. 21, 1403–1407. 10.1016/j.cub.2011.07.021 21820308 PMC3315006

[B41] Ilango A. Kesner A. J. Keller K. L. Stuber G. D. Bonci A. Ikemoto S. (2014). Similar roles of substantia nigra and ventral tegmental dopamine neurons in reward and aversion. J. Neurosci. 34, 817–822. 10.1523/JNEUROSCI.1703-13.2014 24431440 PMC3891961

[B42] Jensen J. L. Marstrand P. C. D. Nielsen J. B. (2005). Motor skill training and strength training are associated with different plastic changes in the central nervous system. J. Appl. Physiol. 99, 1558–1568. 10.1152/japplphysiol.01408.2004 15890749

[B43] Joundi R. A. Jenkinson N. Brittain J.-S. Aziz T. Z. Brown P. (2012). Driving oscillatory activity in the human cortex enhances motor performance. Curr. Biol. 22, 403–407. 10.1016/j.cub.2012.01.024 22305755 PMC3343257

[B44] Kanai R. Chaieb L. Antal A. Walsh V. Paulus W. (2008). Frequency-dependent electrical stimulation of the visual cortex. Curr. Biol. 18, 1839–1843. 10.1016/j.cub.2008.10.027 19026538

[B45] Kanai R. Paulus W. Walsh V. (2010). Transcranial alternating current stimulation (tACS) modulates cortical excitability as assessed by TMS-induced phosphene thresholds. Clin. Neurophysiol. 121, 1551–1554. 10.1016/j.clinph.2010.03.022 20382069

[B46] Kasten E. Bunzenthal U. Sabel B. A. (2006). Visual field recovery after vision restoration therapy (VRT) is independent of eye movements: an eye tracker study. Behav. Brain Res. 175, 18–26. 10.1016/j.bbr.2006.07.024 16970999

[B47] Kasten E. Wüst S. Behrens-Baumann W. Sabel B. A. (1998). Computer-based training for the treatment of partial blindness. Nat. Med. 4, 1083–1087. 10.1038/2079 9734406

[B48] Kourtidou P. Kasselimis D. Potagas C. Zalonis I. Evdokimidis I. (2015). Effects of mental flexibility and motor dysfunction on cognitive performance in patients with parkinson's disease. Arch. Neurosci. 2:e21087 10.5812/archneurosci.21087

[B49] Krause V. Wach C. Südmeyer M. Ferrea S. Schnitzler A. Pollok B. (2013). Cortico-muscular coupling and motor performance are modulated by 20 Hz transcranial alternating current stimulation (tACS) in Parkinson's disease. Front. Hum. Neurosci. 7:928. 10.3389/fnhum.2013.00928 24474912 PMC3893573

[B50] Lesica N. A. Jin J. Weng C. Yeh C.-I. Butts D. A. Stanley G. B.. (2007). Adaptation to stimulus contrast and correlations during natural visual stimulation. Neuron 55, 479–491. 10.1016/j.neuron.2007.07.013 17678859 PMC1994647

[B51] Liebetanz D. Koch R. Mayenfels S. König F. Paulus W. Nitsche M. A. (2009). Safety limits of cathodal transcranial direct current stimulation in rats. Clin. Neurophysiol. 120, 1161–1167. 10.1016/j.clinph.2009.01.022 19403329

[B52] Liu A. Devinsky O. Bryant A. Jefferson A. Friedman D. Shafi M. (2014). Efficacy of transcranial direct current stimulation on working memory and mood in patients with temporal lobe epilepsy (S43.006). Neurology 82, S43006. Available online at: http://www.neurology.org/content/82/10_Supplement/S43.006.short

[B53] Lustenberger C. Boyle M. R. Foulser A. A. Mellin J. M. Fröhlich F. (2015). Functional role of frontal alpha oscillations in creativity. Cortex 67, 74–82. 10.1016/j.cortex.2015.03.012 25913062 PMC4451406

[B54] Mang C. S. Campbell K. L. Ross C. J. D. Boyd L. A. (2013). Promoting neuroplasticity for motor rehabilitation after stroke: considering the effects of aerobic exercise and genetic variation on brain-derived neurotrophic factor. Phys. Ther. 93, 1707–1716. 10.2522/ptj.20130053 23907078 PMC3870490

[B55] Meinzer M. Lindenberg R. Darkow R. Ulm L. Copland D. Flöel A. (2014). Transcranial direct current stimulation and simultaneous functional magnetic resonance imaging. J. Vis. Exp. 86:e51730. 10.3791/51730 24796646 PMC4181345

[B56] Miller J. Berger B. Sauseng P. (2015). Anodal transcranial direct current stimulation (tDCS) increases frontal–midline theta activity in the human EEG: a preliminary investigation of non-invasive stimulation. Neurosci. Lett. 588, 114–119. 10.1016/j.neulet.2015.01.014 25576699

[B57] Miniussi C. Harris J. A. Ruzzoli M. (2013). Modelling non-invasive brain stimulation in cognitive neuroscience. Neurosci. Biobehav. Rev. 37, 1702–1712. 10.1016/j.neubiorev.2013.06.014 23827785

[B58] Miniussi C. Paul W. Rossini P. M. (2012). Transcranial Brain Stimulation. Boca Raton, FL: CRC Press.

[B59] Moliadze V. Antal A. Paulus W. (2010). Boosting brain excitability by transcranial high frequency stimulation in the ripple range. J. Physiol. 588, 4891–4904. 10.1113/jphysiol.2010.196998 20962008 PMC3036186

[B60] Musso M. (1999). Training-induced brain plasticity in aphasia. Brain 122, 1781–1790. 10.1093/brain/122.9.1781 10468516

[B61] Nardone R. Höller Y. Leis S. Höller P. Thon N. Thomschewski A.. (2014). Invasive and non-invasive brain stimulation for treatment of neuropathic pain in patients with spinal cord injury: a review. J. Spinal Cord Med. 37, 19–31. 10.1179/2045772313Y.0000000140 24090372 PMC4066547

[B62] Neuling T. Rach S. Herrmann C. S. (2013). Orchestrating neuronal networks: sustained after-effects of transcranial alternating current stimulation depend upon brain states. Front. Hum. Neurosci. 7:161. 10.3389/fnhum.2013.00161 23641206 PMC3639376

[B63] Nitsche M. A. Paulus W. (2000). Excitability changes induced in the human motor cortex by weak transcranial direct current stimulation. J. Physiol. 527 Pt 3, 633–639. 10.1111/j.1469-7793.2000.t01-1-00633.x 10990547 PMC2270099

[B64] Nitsche M. A. Liebetanz D. Lang N. Antal A. Tergau F. Paulus W. (2003). Safety criteria for transcranial direct current stimulation (tDCS) in humans. Clin. Neurophysiol. 114, 2220–2222; author reply 2222–2223. 10.1016/S1388-2457(03)00235-9 14580622

[B65] Nitsche M. A. Cohen L. G. Wassermann E. M. Priori A. Lang N. Antal A.. (2008). Transcranial direct current stimulation: state of the art 2008. Brain Stimul. 1, 206–223. 10.1016/j.brs.2008.06.004 20633386

[B66] Nizard J. Raoul S. Nguyen J.-P. Lefaucheur J.-P. (2012). Invasive stimulation therapies for the treatment of refractory pain. Discov. Med. 14, 237–246. http://www.discoverymedicine.com/Julien-Nizard/2012/10/23/invasive-stimulation-therapies-for-the-treatment-of-refractory-pain/ 23114579

[B67] Ozen S. Sirota A. Belluscio M. A. Anastassiou C. A. Stark E. Koch C.. (2010). Transcranial electric stimulation entrains cortical neuronal populations in rats. J. Neurosci. 30, 11476–11485. 10.1523/JNEUROSCI.5252-09.2010 20739569 PMC2937280

[B68] Paulus W. (2010). On the difficulties of separating retinal from cortical origins of phosphenes when using transcranial alternating current stimulation (tACS). Clin. Neurophysiol. 121, 987–991. 10.1016/j.clinph.2010.01.029 20181514

[B69] Perrey S. (2013). Promoting motor function by exercising the brain. Brain Sci. 3, 101–122. 10.3390/brainsci3010101 24961309 PMC4061835

[B70] Pogosyan A. Gaynor L. D. Eusebio A. Brown P. (2009). Boosting cortical activity at beta-band frequencies slows movement in humans. Curr. Biol. 19, 1637–1641. 10.1016/j.cub.2009.07.074 19800236 PMC2791174

[B71] Polanía R. Nitsche M. A. Korman C. Batsikadze G. Paulus W. (2012). The importance of timing in segregated theta phase-coupling for cognitive performance. Curr. Biol. 22, 1314–1318. 10.1016/j.cub.2012.05.021 22683259

[B72] Poreisz C. Boros K. Antal A. Paulus W. (2007). Safety aspects of transcranial direct current stimulation concerning healthy subjects and patients. Brain Res. Bull. 72, 208–214. 10.1016/j.brainresbull.2007.01.004 17452283

[B73] Reato D. Rahman A. Bikson M. Parra L. C. (2010). Low-intensity electrical stimulation affects network dynamics by modulating population rate and spike timing. J. Neurosci. 30, 15067–15079. 10.1523/JNEUROSCI.2059-10.2010 21068312 PMC3500391

[B74] Reato D. Rahman A. Bikson M. Parra L. C. (2013). Effects of weak transcranial alternating current stimulation on brain activity-a review of known mechanisms from animal studies. Front. Hum. Neurosci. 7:687. 10.3389/fnhum.2013.00687 24167483 PMC3805939

[B75] Rogde K. Hagen Å. M. Melby-Lervåg M. Lervåg A. Hagen Ã. M. Melby-LervÃ¥g M. (2013). The Effect of Language Comprehension Training on Standardized Tests: A Systematic Review. Available online at: http://campbellcollaboration.org/lib/project/302/ (Accessed November 7, 2014).

[B76] Romanska A. Rezlescu C. Susilo T. Duchaine B. Banissy M. J. (2015). High-frequency transcranial random noise stimulation enhances perception of facial identity. Cereb. Cortex 25, 4334–4340. 10.1093/cercor/bhv016 25662714 PMC4816786

[B77] Sabel B. A. Gudlin J. (2014). Vision restoration training for glaucoma: a randomized clinical trial. JAMA Ophthalmol. 132, 381–389. 10.1001/jamaophthalmol.2013.7963 24504128

[B78] Sabel B. A. Kasten E. (2000). Restoration of vision by training of residual functions. Curr. Opin. Ophthalmol. 11, 430–436. 10.1097/00055735-200012000-00008 11141637

[B79] Sabel B. A. Fedorov A. B. Naue N. Borrmann A. Herrmann C. Gall C. (2011a). Non-invasive alternating current stimulation improves vision in optic neuropathy. Restor. Neurol. Neurosci. 29, 493–505. 10.3233/RNN-2011-0624 22124039

[B80] Sabel B. A. Henrich-Noack P. Fedorov A. Gall C. (2011b). Vision restoration after brain and retina damage: the “residual vision activation theory.” Prog. Brain Res. 192, 199–262. 10.1016/B978-0-444-53355-5.00013-0 21763527

[B81] Santarnecchi E. Brem A.-K. Levenbaum E. Thompson T. Kadosh R. C. Pascual-Leone A. (2015). Enhancing cognition using transcranial electrical stimulation. Curr. Opin. Behav. Sci. 4, 171–178. 10.1016/j.cobeha.2015.06.003

[B82] Santarnecchi E. Polizzotto N. R. Godone M. Giovannelli F. Feurra M. Matzen L.. (2013). Frequency-dependent enhancement of fluid intelligence induced by transcranial oscillatory potentials. Curr. Biol. 23, 1449–1453. 10.1016/j.cub.2013.06.022 23891115

[B83] Schmidt S. Mante A. Rönnefarth M. Fleischmann R. Gall C. Brandt S. A. (2013). Progressive enhancement of alpha activity and visual function in patients with optic neuropathy: a two-week repeated session alternating current stimulation study. Brain Stimul. 6, 87–93. 10.1016/j.brs.2012.03.008 22537864

[B84] Schmoll H. Badan I. Grecksch G. Walker L. Kessler C. Popa-Wagner A. (2003). Kindling status in Sprague-Dawley rats induced by pentylenetetrazole. Am. J. Pathol. 162, 1027–1034. 10.1016/S0002-9440(10)63897-7 12598335 PMC1868098

[B85] Schnitzler A. Gross J. (2005). Normal and pathological oscillatory communication in the brain. Nat. Rev. Neurosci. 6, 285–296. 10.1038/nrn1650 15803160

[B86] Schutter D. J. L. G. Hortensius R. (2010). Retinal origin of phosphenes to transcranial alternating current stimulation. Clin. Neurophysiol. 121, 1080–1084. 10.1016/j.clinph.2009.10.038 20188625

[B87] Schutter D. J. L. G. Hortensius R. (2011). Brain oscillations and frequency-dependent modulation of cortical excitability. Brain Stimul. 4, 97–103. 10.1016/j.brs.2010.07.002 21511210

[B88] Sela T. Kilim A. Lavidor M. (2012). Transcranial alternating current stimulation increases risk-taking behavior in the balloon analog risk task. Front. Neurosci. 6:22. 10.3389/fnins.2012.00022 22347844 PMC3278979

[B89] Sergeeva E. G. Fedorov A. B. Henrich-Noack P. Sabel B. A. (2012). Transcorneal alternating current stimulation induces EEG “aftereffects” only in rats with an intact visual system but not after severe optic nerve damage. J. Neurophysiol. 108, 2494–2500. 10.1152/jn.00341.2012 22875900

[B90] Shah P. P. Szaflarski J. P. Allendorfer J. Hamilton R. H. (2013). Induction of neuroplasticity and recovery in post-stroke aphasia by non-invasive brain stimulation. Front. Hum. Neurosci. 7:888. 10.3389/fnhum.2013.00888 24399952 PMC3870921

[B91] Terney D. Chaieb L. Moliadze V. Antal A. Paulus W. (2008). Increasing human brain excitability by transcranial high-frequency random noise stimulation. J. Neurosci. 28, 14147–14155. 10.1523/JNEUROSCI.4248-08.2008 19109497 PMC6671476

[B92] Vedam-Mai V. Gardner B. Okun M. S. Siebzehnrubl F. A. Kam M. Aponso P.. (2014). Increased precursor cell proliferation after deep brain stimulation for Parkinson's disease: a human study. PLoS ONE 9:e88770. 10.1371/journal.pone.0088770 24594681 PMC3940428

[B93] Vossen A. Gross J. Thut G. (2015). Alpha power increase after transcranial alternating current stimulation at alpha-frequency (α-tACS) reflects plastic changes rather than entrainment. Brain Stimul. 8, 499–508. 10.1016/j.brs.2014.12.004 25648377 PMC4464304

[B94] Wach C. Krause V. Moliadze V. Paulus W. Schnitzler A. Pollok B. (2013). The effect of 10 Hz transcranial alternating current stimulation (tACS) on corticomuscular coherence. Front. Hum. Neurosci. 7:511. 10.3389/fnhum.2013.00511 24009573 PMC3756226

[B95] Wang H.-P. Spencer D. Fellous J.-M. Sejnowski T. J. (2010). Synchrony of thalamocortical inputs maximizes cortical reliability. Science 328, 106–109. 10.1126/science.1183108 20360111 PMC2859205

[B96] Wirth M. Rahman R. A. Kuenecke J. Koenig T. Horn H. Sommer W.. (2011). Effects of transcranial direct current stimulation (tDCS) on behaviour and electrophysiology of language production. Neuropsychologia 49, 3989–3998. 10.1016/j.neuropsychologia.2011.10.015 22044650

[B97] Zaehle T. Rach S. Herrmann C. S. (2010). Transcranial alternating current stimulation enhances individual alpha activity in human EEG. PLoS ONE 5:e13766. 10.1371/journal.pone.0013766 21072168 PMC2967471

[B98] Zaghi S. Acar M. Hultgren B. Boggio P. S. Fregni F. (2010). Noninvasive brain stimulation with low-intensity electrical currents: putative mechanisms of action for direct and alternating current stimulation. Neuroscientist 16, 285–307. 10.1177/1073858409336227 20040569

